# Neurons Induced From Fibroblasts of c9ALS/FTD Patients Reproduce the Pathology Seen in the Central Nervous System

**DOI:** 10.3389/fnins.2019.00935

**Published:** 2019-09-06

**Authors:** Peter O. Bauer, Judith H. Dunmore, Hiroki Sasaguri, Vaclav Matoska

**Affiliations:** ^1^Department of Neuroscience, Mayo Clinic, Jacksonville, FL, United States; ^2^Bioinova, Ltd., Prague, Czechia; ^3^Department of Clinical Biochemistry, Hematology and Immunology, Na Homolce Hospital, Prague, Czechia

**Keywords:** amyotrophic lateral sclerosis, c9ALS/FTD, c9orf72 expansion, induced neurons, iNeurons, RNA foci, repeat-associated non-ATG translation, anti-sense oligonucleotides

## Abstract

Amyotrophic lateral sclerosis (ALS) and frontotemporal dementia (FTD) are incurable neurodegenerative conditions. A non-coding hexanucleotide (GGGGCC) repeat expansion in the *c9orf72* gene is the most common genetic cause of ALS/FTD. We present a cellular model of c9ALS/FTD where induced neurons (iNeurons) are generated within 2 weeks by direct conversion of patients‘ dermal fibroblasts through down-regulation of polypyrimidine-tract-binding protein 1 (PTB1). While sense (S) and anti-sense (AS) intranuclear RNA foci were observed in both fibroblasts and iNeurons, the accumulation of (S) and (AS) repeat-associated non-ATG translation (RANT) products were detected only in iNeurons. Importantly, anti-sense oligonucleotides (ASOs) against the (S) repeat transcript lead to decreased (S) RNA foci staining and a reduction of the corresponding RANT products without affecting its (AS) counterparts. ASOs treatment also rescued the cell viability upon stressful stimulus. The results indicate that iNeurons is an advantageous model that not only recapitulates c9ALS/FTD hallmark features but can also help uncover promising therapeutics.

## Introduction

Amyotrophic lateral sclerosis (ALS) and frontotemporal dementia (FTD) are devastating neurodegenerative diseases with significant genetic, neuropathological, and clinical overlap (Lillo and Hodges, 2009; Walsh and Hochedlinger, 2010). ALS is characterized by selective degeneration of upper and lower motor neurons causing muscle weakness, spasticity, atrophy, and death within 1–5 years after disease onset (Cleveland and Rothstein, 2001; Bruijn et al., 2004). FTD is one of the most common early onset dementias (Ratnavalli et al., 2002) characterized by neuronal degeneration in the frontal and temporal lobes causing a progressive deterioration of behavior, language, and personality (Bozeat et al., 2000). An expanded non-coding hexanucleotide repeat expansion [r(GGGGCC)_exp_] in the chromosome 9 open reading frame 72 (*c9orf72*) gene has been identified as the most common genetic cause underlying FTD and ALS (c9ALS/FTD) (DeJesus-Hernandez et al., 2011; Renton et al., 2011). Normal length is considered less between 2 and 30 hexanucleotides while 65 repeats are regarded as the limit for the pathogenic range [r(GGGGCC)_exp_] (Loy et al., 2014; Van Mossevelde et al., 2017). Approximately 40% of FTD patients develop motor neuron dysfunction (MND) (Lomen-Hoerth et al., 2003) and as approximately 15% of ALS patients also meet the criteria for FTD (Giordana et al., 2011; Phukan et al., 2012) while up to 50% of cases displaying frontotemporal dysfunction features (Montuschi et al., 2015).

The repeat expansion may cause c9ALS/FTD through three potential pathomechanisms. Firstly, RNA transcripts containing the expansion of sense (S) r(GGGGCC)_exp_ or anti-sense (AS) r(CCCCGG)_exp_ can form nuclear foci in the CNS of c9ALS/FTD patients (Gendron et al., 2013; Lagier-Tourenne et al., 2013; Taylor et al., 2016; Liu et al., 2017) which may lead to RNA-mediated toxicity by sequestering RNA-binding proteins (RBP) impairing their ability to interact with their physiological RNA targets (Lee et al., 2013; Gendron et al., 2014). Secondly, (S) and (AS) RNA transcripts can be processed by repeat associated non-ATG translation (c9RANT) resulting in dipeptide repeat proteins (S: poly GP, GA, GR; AS: poly PR, PA, GP) production that are prone to form neuronal inclusions in the CNS (Ash et al., 2013; Gendron et al., 2013; Mori et al., 2013). Thirdly, reduction of *c9orf72* mRNA levels in brain and other tissues of repeat expansion carriers were first reported in 2011 (DeJesus-Hernandez et al., 2011; Renton et al., 2011) in which several other groups and we subsequently have reported this reduction may largely due to histones hypermethylation in the vicinity of the *c9orf72* gene (Belzil et al., 2013), indicating that loss of yet unknown function of the C9orf72 protein may also play a role in disease pathogenesis (Belzil et al., 2014; Bauer, 2016; Shi et al., 2018). The haploinsufficiency of the C9orf72 protein, however, seems to be not sufficient to cause the disease, as has been shown in a C9orf72 knock out mouse model (Koppers et al., 2015; Sudria-Lopez et al., 2016). Nevertheless, it is thought to affect the inflammatory response during the disease course (O’Rourke et al., 2016).

Several cellular models of c9ALS/FTD have been generated, however, most of them have limitations. For example, although overexpression of the expansion results in robust RNA foci formation and c9RANT products accumulation (Wen et al., 2014; Molliex et al., 2015; Webster et al., 2016; Gendron et al., 2017), it does not result in reduced expression of *c9orf72* mRNA and protein as seen in c9ALS/FTD patients (DeJesus-Hernandez et al., 2011; Renton et al., 2011). It may also bypass potentially important events resulting from the presence of the repeat in genomic DNA. Moreover, it is very challenging to clone repeat sequences with lengths similar to those in patients.

So far, the most accurate cellular models have been considered neurons generated from induced pluripotent stem cells (iPSCs) derived from c9ALS/FTD patients. In these cells, RNA foci and accumulation of inclusions composed of c9RAN proteins, mainly poly GP, have been observed while maintaining the reduction of *C9orf72* mRNA (Almeida et al., 2013; Donnelly et al., 2013; Sareen et al., 2013). Although robust, the preparation of this type of cell model can be costly and time-consuming. Here we present a method involving direct conversion of human dermal fibroblasts (HDFs) into induced neurons (iNeurons). We used a previously described vector encoding short hairpin RNA targeting polypyrimidine-tract-binding protein 1 (PTB1) (Xue et al., 2013). PTB1 has been shown to inhibit neuronal differentiation triggered by miR-124 while miR-124 can reduce PTB1 resulting in a cascade including proneuronal alternative splicing events (Makeyev et al., 2007). Xue et al. (2013) generated neurons from different types of cells including HeLa, NT2, Neuro2a, or mouse embryonic fibroblasts (Xue et al., 2013). We developed a modified protocol for direct transformation of HDFs to iNeurons within 2 weeks compared to 12–16 weeks through conventional iPSC differentiation. Characterization of these iNeurons generated from c9ALS/FTD patients showed expression of all six (S) and (AS)-c9RANT products detected by polyclonal antibodies against each individual protein addition to the presence of intranuclear (S) and (AS) RNA foci in all tested cell lines. Moreover, the usefulness of iNeurons in *in vitro* modeling of ALS has been shown recently (Lim et al., 2016). In this study, iNeurons generated from fibroblasts of three ALS patients with mutations in fused in sarcoma (FUS) gene recapitulated all key features of FUS pathology found in the patient brain and spinal cord motor neurons.

Several groups have recently shown that anti-sense oligonucleotides (ASOs) therapy targeting the r(GGGGCC)_exp_ RNA sequence in iPSC differentiated neurons can significantly reduce (S) RNA foci and mitigate cellular toxicity with little effect on c9RAN products detected with a polyclonal antibody preferentially detecting poly GP (Donnelly et al., 2013; Sareen et al., 2013). Therefore, we tested antisense oligonucleotides (ASOs) targeting the sense transcript containing repeat expansion r(GGGGCC)_exp_ in our model and observed significant reduction of (S) RNA foci and c9RANT products accumulation without affecting (AS) RNA foci and c9RANT products. Thus, our c9ALS/FTD iNeuron model may be an efficient tool for validating therapeutic screenings.

## Materials and Methods

### Standard Protocol Approvals, Registrations, and Patient Consents

Protocols were approved by the Mayo Clinic Institutional Review Board and Ethics Committee on human experimentation. Skin biopsies were collected after the participants gave written informed consent.

### Human Samples

Punch skin biopsies were performed on the anterior aspect of the forearm from six individuals visiting Department of Neurology of Mayo Clinic, which included three control participants (control 1: female diagnosed with sixth nerve palsy, 61 years old at the time of biopsy; control 2: healthy female, 64 years; control 3: healthy female, 38 years) and three repeat expansion carriers (carrier 1: female, asymptomatic, 28 years old; carrier 2: female diagnosed with ALS at 49 years of age, 50 years; carrier 3: male diagnosed with ALS/FTD at 41 years of age, 43 years). Fibroblasts were generated by ReGen Theranostics Inc., (Rochester, MN). The presence or absence of the *c9orf72* repeat expansion was determined by PCR method as previously described (DeJesus-Hernandez et al., 2011). These studies were approved by Institutional Review Board and all participants provided written informed consent.

### Cell Culture and Differentiation of iNeurons

Fibroblasts were maintained in Dulbecco’s modified Eagle’s medium (DMEM; Lonza) supplemented with 10% heat-inactivated fetal bovine serum (Sigma), 100 units/ml penicillin, and 100 μg/ml streptomycin (Gibco) at 37°C, in an atmosphere containing 5% CO2 and 95% air. The shRNA against human PTB1 (shPTB) cloned into lentiviral plasmid pLKO.1 was a kind gift from Dr. Fu (University of California, San Diego, CA, United States). Both shPTB and non-silencing shRNA in the pLKO.1 vector (Sigma) were packaged in HEK293FT cells using Virapower (Invitrogen). Viral particles were collected 48 and 72 h after transfection.

For iNeurons production, our previously modified method (Su et al., 2014) was further optimized. Fibroblasts were seeded on a poly-D-lysine-coated surface and were transduced with lentiviral particles with shPTB for 16–18 h in the presence of 5 μg/ml polybrene. We selected the infected cells with 1.5 μg/ml puromycin for 48 h starting 2 days after transduction. At day 5, 10 ng/ml basic fibroblast growth factor (bFGF; GenScript) was added to the medium for 2 days. Cells were then maintained in DMEM/F12 medium containing 5% FBS (reduced to 2% after 2 days), 25 mg/ml insulin, 100 nM putrescine, 50 mg/ml transferrin, 30 nM sodium selenite (all Sigma), and 15 ng/ml bFGF. After 4 days, we added B27 supplement (Gibco) and 10 ng/ml each of brain-derived neurotrophic factor (BDNF), glial cell-derived neurotrophic factor (GDNF; both R&D Systems), neurotensin-3 (NT3; Peprotech), and ciliary neurotrophic factor (CNTF; Sigma). The cells were used for experiments 2–4 days later.

### Immunocytochemistry

Cells were fixed with 4% paraformaldehyde, permeabilized with 0.5% Triton X-100/PBS and blocked with 5% skim milk in TBS-Tween20 (TBS-T) buffer. All antibodies were diluted in 5% skim milk/TBS-T. For neuronal markers analysis, following antibodies were used: mouse anti-MAP2 (Sigma; 1:2,000 dilution), mouse anti-Neurofilament H (Smi-32; 1:2,000), mouse anti-neuronal nuclei (NeuN; 1:500), rabbit anti-Synapsin 1 (Syn1; 1:500), rabbit anti-MAP2 (all Millipore; 1:2,000), mouse anti-Tuj1 (Cell Signaling, 1:2,000), rabbit anti-postsynaptic density protein 95 (PSD95; 1:250), rabbit anti-Drebrin (both Abcam; 1:500), rabbit anti-pan voltage-gated Na2 + channels (pan Nav; Alomone; 1:250), anti-vesicular glutamate transporter 1 (vGlut1; Synaptic Systems; 1:500), and goat polyclonal anti-PTB1 (Abcam; 1:200). Rabbit antibodies against c9RANT products, anti-poly(GP), anti-poly(GA), anti-poly(GR), anti-poly(PA), and anti-poly(PR), were generated as described previously (Gendron et al., 2013; Mann et al., 2013) and used at 1:1,000 dilutions. Secondary fluorescent antibodies (Invitrogen) were used at 1:1000 dilutions. Nuclei were stained with Hoechst 33258 (Invitrogen). Confocal microscopy was performed using Zeiss LSM 510 microscope.

### Western Blot Analysis

Fibroblasts were transduced with shPTB1 or non-silencing control shRNA. Cells were collected and lysed 5 days later and the samples were run on 4–20% Tris–glycine polyacrylamide gel. Proteins were then transferred from the gel to nitrocellulose membranes following standard protocols and the immunoblot was completed using goat polyclonal anti-PTB1 (1:1,000) and mouse monoclonal anti-β-actin (Sigma; 1:10,000).

### Cell Treatment

Control anti-sense oligonucleotides (ctrl ASO; sequence: CCTTCCCTGAAGGTTCCTCC) and ASOs targeting the r(GGGGCC)_exp_ (ASOs; sequence: CCGGCCCCGGCCCCGGCCCC) (Donnelly et al., 2013) in final concentration of 5 μM were added to the cells at day 5, after the puromycin selection. Modified 2′ *O*-methyl RNA/DNA ASOs were generated by Integrated DNA Technologies (IDT). For quantification experiments, iNeurons were induced in 96-well plates and serial pictures were generated with BD Pathway Bio-imager (BD Biosciences). For each cell line and condition, the percentage of cells containing c9RANT inclusions was calculated from 6 wells in 4 independent experiments.

### RNA Fluorescence *in situ* Hybridization (FISH)

RNA FISH on fibroblasts and iNeurons to detect RNA foci consisting of the sense (S) transcript was performed using a modified method described previously (Lagier-Tourenne et al., 2013). Briefly, cells on coverslips were fixed in 4% PFA/DEPC-PBS, permeabilized with 0.2% Triton X-100/DEPC-PBS and washed twice with DEPC-PBS. The samples were then dehydrated by 70 and 100% ethanol and air dried. This step was followed by incubation in hybridization buffer (10% dextran sulfate, 50% formamide, 50 mM sodium phosphate buffer (pH 7), 2xSSC) at 66°C for 20–60 min. Prior to use, the locked nucleic acid (LNA) probe (5′TYE563-CCCCGGCCCCGGCCCC-3′, Batch #612968, Exiqon) was denatured at 80°C for 75 s and diluted to 40 nM with hybridization buffer. The hybridization with probe was performed in a sealed, light-protected chamber at 66°C for 16–24 h. RNA FISH of fibroblasts and iNeurons to detect RNA foci consisting of the (AS) transcript were performed as above with slight modifications. The incubation with hybridization buffer (10% dextran sulfate, 50% formamide, 50 mM lithium phosphate buffer (pH 7), 2xSSC) changed to 60°C for 16–24 h with LNA probe (5′TYE563-GGGGCCGGGGCCGGGG, batch #614784, Exiqon). The coverslips were subsequently washed with 0.1% Tween-20/2xSSC for 5 min followed by three 10 min stringency washes in 0.1xSSC at 66°C. The cells were stained with Hoechst 33258, rinsed with DEPC-treated water, dehydrated through 70 and 100% ethanol and air dried. Coverslips were then mounted with Prolong Gold anti-fade reagent (Life Technologies) and the RNA foci were visualized and quantified using a Zeiss Axiovert Fluorescence Microscope with apotome module. For each cell line, three experiments were performed with three fields consisting of at least 100 cells each were randomly selected per condition. For each field, the number of foci-positive nuclei and the total number of nuclei were counted to determine the average percentage of foci-positive cells.

### Cell Toxicity Assay

The iNeurons were grown in 96-well plates and treated with increasing concentrations of tunicamycin (Sigma) for 24 h or 10 μM glutamate (Sigma) for 8 h. Medium was collected and lactate dehydrogenase (LDH) levels using Cyto-tox 96 (Promega) were measured with Spectra Max M5e Microplate Reader (Molecular Devices).

### Statistical Analysis

We used paired student’s *t*-test for comparison between two sample groups. Two-way ANOVA Fisher’s test was used for multiple comparisons with a 95% confidence level. We considered the difference between comparisons to be significant when *P* < 0.05 for all the statistical analyses.

## Results

### Knock-Down of PTB1 in Human Dermal Fibroblasts Leads to Induction of Neural Phenotype

**FIGURE 1:**
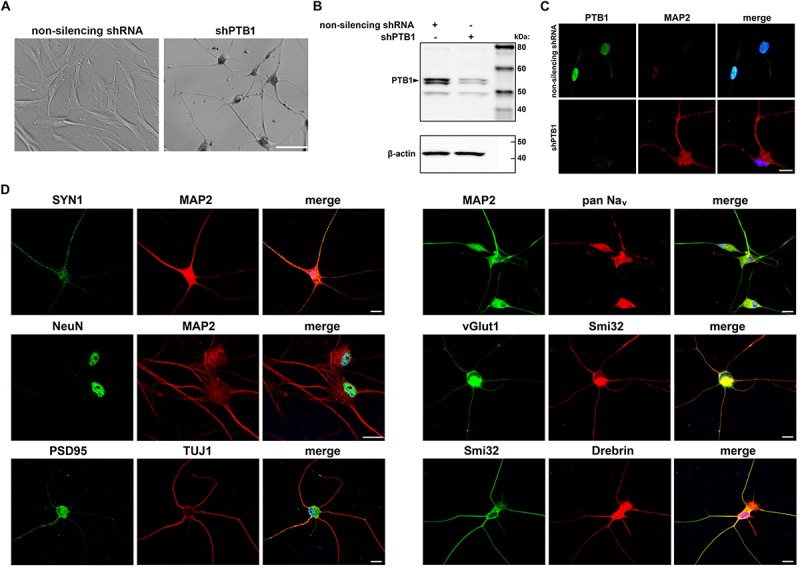
Generation and characterization of c9ALS/FTD patient-specific induced neurons (iNeurons). **(A)** Representative bright field images show cell morphology upon non-silencing shRNA and shPTB1 transduction of human fibroblasts. While the cells infected with control shRNA retained their fibroblast-like shape, shPTB1-transduction induced a neuronal morphology with reduced size of cell soma, and neurite outgrowth. Scale bar, 400 μm. **(B)** Western blot analysis of fibroblasts transduced or not with shPTB1. **(C)** Immunofluorescence staining of cells transduced with non-silencing shRNA or shPTB1 for PTB1 and the neuronal marker, MAP2. Scale bar, 20 μm. **(D)** Expression of cytoskeletal (MAP2, TUJ1, Smi32), synaptic (SYN1, PSD95) and other neuronal markers in iNeurons. These cells also express Drebrin, which plays a role in the formation and maintenance of dendritic spines in neurons. Scale bars, 20 μm.

Effect of PTB1 downregulation in several cell types has been shown to result in direct conversion to neuron-like cells (Xue et al., 2013). In an attempt to develop human neuronal disease models with endogenous mutation, we extended this finding to adult human dermal fibroblasts (HDFs). HDFs obtained by skin biopsies were transduced with pLKO.1-shPTB1 packaged into lentiviral particles. Interestingly, cells started to acquire neuron-like morphology as early as 4 days after transduction and with further differentiation process, the cells continued to develop into iNeurons (Figure 1A). The iNeurons with down-regulated PTB1 (Figure 1B) also expressed neuronal marker MAP2 (Figure 1C). In addition, differentiated cells expressed multiple neuron-specific markers including cytoskeletal, synaptic, nuclear, and other markers after 12–14 days after transduction (Figure 1D). Of note, HDFs transduced with non-silencing shRNA did not exhibit neurite outgrowth or neuronal markers expression.

### iNeurons Derived From c9ALS/FTD Patients Form RNA Foci and Accumulate RANT Products

**FIGURE 2:**
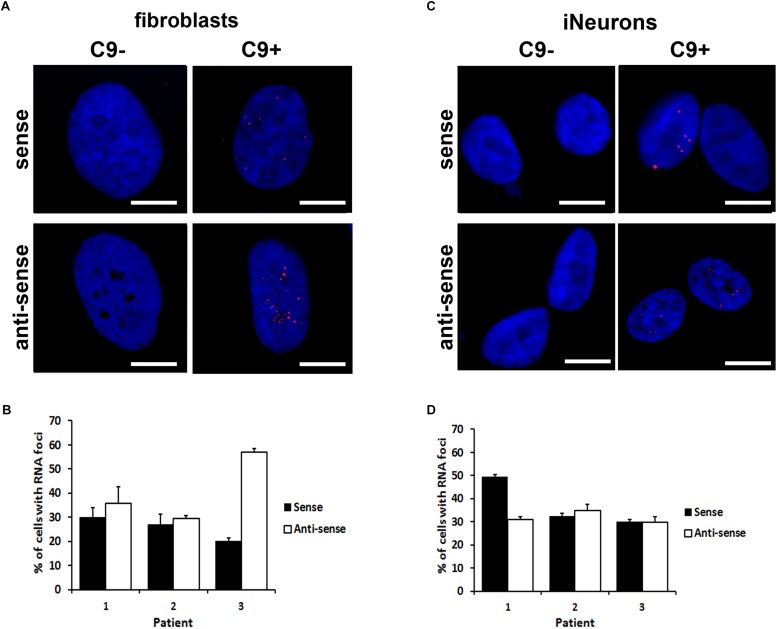
Formation of sense and anti-sense (AS) RNA foci. **(A)** RNA foci in fibroblasts. Blue staining: Hoechst 33258. Scale bars, 20 μm. **(B)** Quantification of fibroblasts with RNA foci. Data represents mean ± standard deviation (SD; *n* = 3, see “Materials and Methods”). **(C)** RNA foci in iNeurons. Blue staining: Hoechst 33258. Scale bars, 20 μm. **(D)** Quantification of iNeurons with RNA foci. Data represents mean ± standard deviation (SD; *n* = 3, see “Materials and Methods”).

To see whether iNeurons could serve as a useful cellular model for *c9orf72*-related pathologies, we analyzed them for the presence of RNA foci, usually found in c9ALS/FTD patients. As reported previously, HDFs of c9ALS/FTD patients contain intranuclear RNA foci (Lagier-Tourenne et al., 2013). (S) and (AS) foci were detected in both fibroblasts and iNeurons by RNA fluorescent *in situ* hybridization (RNA FISH) by utilizing fluorescently labeled locked nucleic acid (LNA) oligonucleotides probes against the (S) and (AS) repeat RNA expansion (Figure 2). RNA foci were not detected in non-c9orf72 (C9-) HDF cell lines. The fraction of cells containing intranuclear (S) foci varied between ∼20% and 30% in HDFs whereas after differentiation into iNeurons, the fraction of cells containing (S) foci varied between ∼30 and 50% among the three c9ALS/FTD patient cell lines (Figures 2C,D). Interestingly Patient 3 had more than 50% of the cells accumulating intranuclear (AS) RNA foci in HDFs (Figure 2C) but is reduced by half in iNeurons. Majority of the c9ALS/FTD fibroblasts and iNeurons had 1 to 4 (S) or (AS) foci per cell with less than 5% exhibiting more than 10 nuclear foci (Supplementary Figure 1). The percentage of cells with 5–10 (S) foci/cell increased whereas the numbers of >20 (AS) foci/cell decreased in iNeurons compared to HDFs (Supplementary Figure 1). The variability of RNA foci observed in our three iNeurons cell lines generated from *c9orf72* expansion carriers (C9 +) are consistent with results previously reported in iPSC derived neurons from *c9orf72* ALS fibroblasts (Donnelly et al., 2013; Sareen et al., 2013).

**FIGURE 3:**
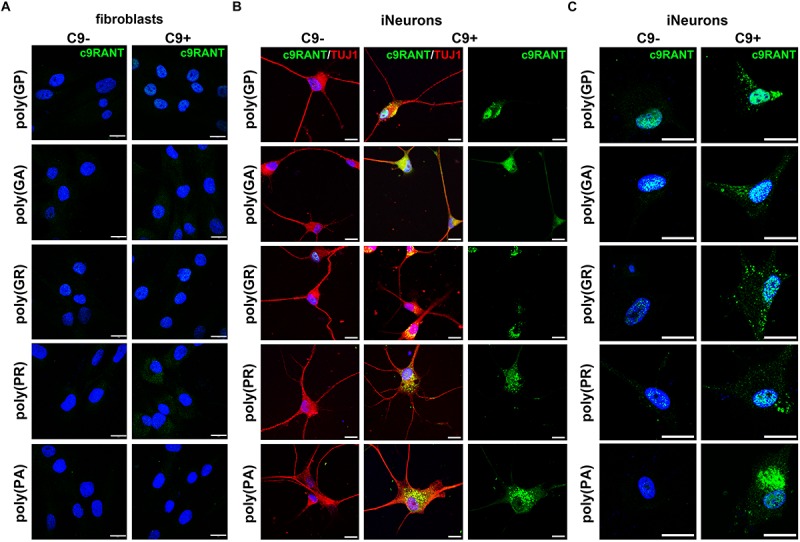
iNeurons express c9RANT products. **(A)** c9ALS/FTD patients’ fibroblasts do not accumulate RANT peptides. **(B)** Accumulation of c9RANT products can be detected in iNeurons generated from c9ALS/FTD patients. **(C)** Distribution of c9RANT products in iNeurons generated from c9ALS/FTD patients. Blue staining: Hoechst 33258. Scale bars, 20 μm.

In contrast to the RNA foci, we were not able to detect RANT products in HDFs (Figure 3A). We were curious whether we could detect some of the RANT products in iNeurons. Surprisingly, we observed all RANT products from the (S) [poly-(GA) and -(GR)], or (AS) repeat [poly-(PR) and -(PA)]; poly-(GP) is translated from both strands (Figure 3B). Interestingly, different RANT peptides displayed distinct distribution and pattern of inclusions (Figure 3C). Moreover, partial translocation of TAR DNA-binding protein 43 (TDP-43) from its normal nuclear location to cytoplasm of the C9 + iNeurons was observed (Supplementary Figure 2). These results indicate that C9 + iNeurons recapitulate main pathological hallmarks of c9ALS/FTD and can be used as an accurate cellular model obtainable quickly and easily.

### RNA Foci Staining and RANT Is Reduced by Antisense Oligonucleotides Treatment

To examine, whether our iNeuron model could be utilized as a tool for research related to experimental therapies, we treated the cells with ASOs. Specifically designed antisense oligonucleotides have been proven to mediate the target RNA molecules degradation through RNase H-based mechanism. Previously, it has been shown that a 3-day treatment with ASOs efficiently reduced the RNA foci formation, however, did not significantly affect poly(GP) accumulation (Donnelly et al., 2013).

**FIGURE 4:**
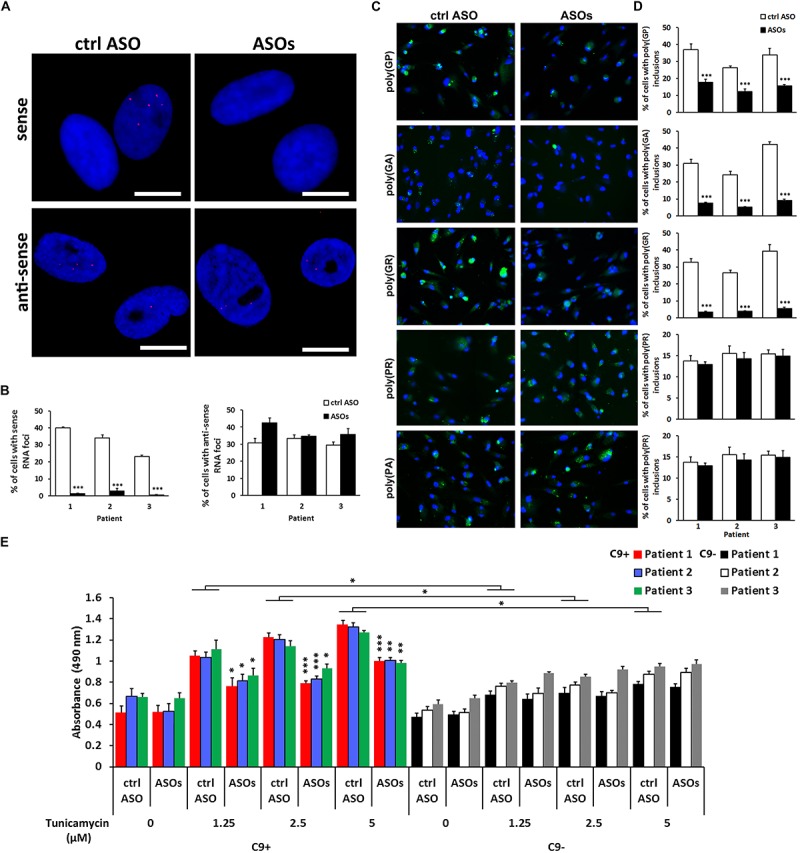
Effect of anti-sense oligonucleotides(ASOs) targeting the sense strand of the c9orf72 repeat, r(GGGGCC)_exp_, on RNA foci staining, c9RANT products accumulation and cell viability. **(A)** ASOs reduced the number of iNeurons with visualized sense but not anti-sense RNA foci. Magnification, 20 ×. **(B)** Quantification of iNeurons containing RNA foci. Data represents mean ± standard error of the mean (SEM; *n* = 4, each with at least 300 cells from 12 wells in 96-well plates). (^∗∗∗^*p* < 0.001). **(C)** Treatment with ASOs reduced the number of cells with inclusions formed by sense RANT peptides: poly(GP),(GA), and (GR) while products translated only from the anti-sense strand, poly(PR) and (PA), remained unchanged. Blue staining: Hoechst 33258. Scale bars, 20 μm. **(D)** Quantification of iNeurons containing inclusions formed by RANT products. Data represents mean ± standard deviation (SD; *n* = 3). (^∗∗∗^*p* < 0.001). **(E)** ASOs reduce the dose-dependent tunicamycin toxicity in C9 + iNeurons. Data represents mean ± standard error of the mean (SEM; *n* = 4, each with data from two wells). (^∗^*p* < 0.05, ^∗∗^*p* < 0.005, ^∗∗∗^*p* < 0.001).

Control ASO or ASOs (5 μM) were applied to the cells 4 days post-transduction. Further differentiation steps including the media changes remained unmodified. ASOs reduced the staining of RNA foci formed from the (S) strand by more than 90% in three different C9 + iNeuron lines (Figures 4A,B). The RNA foci staining from the (AS) strand, on the other hand, was not inhibited (Figures 4A,B). Similar effects of ASOs were observed in the fibroblasts (Supplementary Figure 3).

Importantly, we also observed effect on c9RANT products translated from the (S) strand. The quantification was performed by counting cells containing inclusions by BD Pathway Bio-imager. In case of poly(GR) and poly(GA) peptides, the peptides translated only from the (S) strand, the inclusion formation was reduced by more than 85 and 75%, respectively, in all three C9 + iNeuron lines (Figures 4C,D). Accordingly to (AS) RNA foci, the inclusion formed by (AS) c9RANT products were not affected by ASOs treatment. Poly(GP) inclusions were reduced only by about 52–54% likely due to the fact that it is translated from both (S) and (AS) repeat sequence.

Solely the presence of the r(GGGGCC)_exp_ did not cause cytotoxicity in iNeurons. However, the C9 + cells were more sensitive to the treatment with a well-known endoplasmic reticulum stress inducer, tunicamycin (Figure 4E and Supplementary Figure 4). This is similar to a previous study showing no difference in cell viability in iPSC-derived neurons with or without r(GGGGCC)_exp_. The C9 + iPSC-derived neurons were, however, more sensitive to the proteotoxic stress (Haeusler et al., 2014). Importantly, ASOs treatment was able to reduce the toxicity of tunicamycin, as determined by LDH test and by propidium iodide staining (Figure 4E and Supplementary Figure 4). Similar effect of ASOs was observed in C9 + iNeurons treated with glutamate (Supplementary Figure 5), that has previously been shown to be more toxic to C9 + iPSC-derived neurons (Donnelly et al., 2013). Our results suggest, that blocking the (S) RNA strand leading to reduction of c9RANT products accumulation represents a promising therapeutic strategy for c9ALS/FTD.

These results confirm that iNeurons are a suitable cellular disease model system for screening and performing experimental therapies for c9ALS/FTD.

## Discussion

In this study, we characterize a cellular model of c9ALS/FTD and provide evidence that this model is suitable for experimental therapeutic attempts. Studying neurological diseases is very challenging as compared to other medical fields due to the inaccessibility of the CNS neurons affected by diseases. Most of the human brain studies in neurological patients have been performed on post-mortem tissues often depicting the end-stage of the disease. With improving cellular and animal models of the GGGGCC repeat expansion, we can uncover the pathomechanism related to c9ALS/FTD. Traditional approaches in modeling disease have multiple limitations often due to overexpression of the disease gene. Moreover, cellular and animal models often partially imitate the disease leading to limited phenotypic correlations between the genetic models and human diseases. Consequently, most of the successful experimental treatments fail to be introduced to clinics.

New approaches in disease modeling are not only vital to recapitulate human neuropathologic conditions but also can reveal unknown aspects, which could greatly improve the benefits for patients. It is particularly true in disorders without definitive causative genes and mutations (e.g., sporadic cases of AD, PD, ALS) as well as in sporadic neurological disorders caused by interactions of environmental and (multi)genetic risk factors. Another category comprises disorders where the cloning of the causing genes is problematic (e.g., CG-rich sequence, repeat expansions). The cell reprograming technology for producing iPSCs allows studying the development and progression of neurological diseases in human systems with the disease-specific pathways investigated before and during disease onset. Several reports have been published on employing iPSC technology in modeling neurological diseases, including PD, AD, spinal muscular atrophy or familial dysautonomia (Yung et al., 2013).

Direct conversion of somatic cells bypassing the iPSC stage is an alternative approach which considerably shortens the process patient-specific neurons generation. There have been several methods published using various cocktails of transcription factors (e.g., Ascl1, Brn2, or Myt1l, etc.) (Qiang et al., 2013). These methods appear to be more efficient with as much as 10% of cells conversion rate when compared with those involving iPSC generation (Pfisterer et al., 2011). The strategy of direct conversion has been even more simplified after the discovery of miR-124 (a microRNA highly expressed in neurons which plays an important role in neural development) being able to efficiently contribute to induction of neurons (Ambasudhan et al., 2011) and that the down-regulation of PTB1 by shRNA, leading to an activation of miR-124-related proneuronal expression cascade, was sufficient to transdifferentiate multiple cell lines to neurons (Xue et al., 2013). The delivery of a single factor to the cells enables an extremely simple strategy in changing the fate of differentiated cells.

We extended this finding and used shPTB1 in HDFs obtained from c9ALS/FTD patients. Within 2 weeks, we were able to generate iNeurons which expressed multiple neuronal markers (Figure 1). Importantly, iNeurons displayed both main pathological hallmarks seen in c9ALS/FTD patients: accumulation of RNA foci and c9RANT products (Figures 2, 3). Inclusions formed by c9RANT products were present only in the C9 + iNeurons while not detected in the C9 + fibroblasts (Figure 3). These findings are consistent with our prior observation that poly(GP) inclusions are restricted to neurons in c9ALS/FTD patients (Ash et al., 2013). We also observed a partial translocation of TDP-43 from nucleus to the cytoplasm in the C9 + iNeurons (Supplementary Figure 2). The presence of the repeat expansion did not appear to interfere with the neuronal differentiation process and did not lead to decreased viability of the iNeurons. With additional stressful stimuli, the vulnerability of iNeurons was exposed and displayed increased cytotoxicity associated with r(GGGGCC)_exp_ (Figure 4E). To test the suitability of our model for experimental therapeutic testing, we treated the cells with ASOs targeting the (S) repeat sequence (Donnelly et al., 2013). We found that ASOs the c9RANT products accumulation (Figures 4A–D). Importantly, the viability of the ASOs-treated C9 + iNeurons also increased after treatment with tunicamycin (Figure 4E and Supplementary Figure 4) and glutamate (Supplementary Figure 5).

Reduction of the *c9orf72* mRNA has been shown to be one of the pathological hallmarks of the c9ALS/FTD disease (DeJesus-Hernandez et al., 2011; Renton et al., 2011), probably affecting the inflammatory response (O’Rourke et al., 2016). We have previously observed such reduction in fibroblasts from patients with *c9orf72* mutation, including those involved in this study (Belzil et al., 2013).

The simplicity of the direct conversion strategy enables rapid production of iNeurons, as the whole procedure routinely takes 2–3 weeks. As the primary fibroblast cultures from punch biopsies can be established in 4–6 weeks, the patient-specific iNeurons can be generated within 2 months after the patient’s visit. Therefore, this model represents a powerful tool not only for accurately recapitulating the pathology of c9ALS/FTD but also for individualized testing of potentially effective drugs in the future.

## Author Contributions

POB conceived and designed the experiments. POB, JHD, and HS performed the experiments. POB andVManalyzed the data. POB and VM wrote the manuscript.

## Conflict of Interest Statement

POB is employed by the company Bioinova, Ltd.

The remaining authors declare that the research was conducted in the absence of any commercial or financial relationships that could be construed as a potential conflict of interest.
