# Supplementary material for: Neurons Induced From Fibroblasts of c9ALS/FTD Patients Reproduce the Pathology Seen in the Central Nervous System
Source: Front Neurosci. 2019 Sep 6;13:935. doi: 10.3389/fnins.2019.00935 (PMC6743368; doi:10.3389/fnins.2019.00935)
Supplement: Supplementary file 1 [file Data_Sheet_1.pdf]

Supplementary Information

Neurons induced from fibroblasts of c9ALS/FTD patients reproduce the pathology seen in the central nervous system

Peter O. Bauer, Judith H. Dunmore, Hiroki Sasaguri, Vaclav Matoska

Supplementary Material and Methods

Immunocytochemistry

Cells were processed as described in Material and Methods. Rabbit anti-TDP-43 (Proteintech; 1:1000) and mouse anti-ALYREF (Thermo Fisher; 1:1000) antibodies were diluted in 5% skim milk/TBS-T.

Cell toxicity assay

The iNeurons were grown in 96-well plates and treated with 5 μM of tunicamycin. After 24 hrs, 5μg/ml each of Hoechst 33258 (Invitrogen) and propidium iodide (PI) were added to the cells followed by 10 min incubation at 37°C. The PI-positive cells were quantified using BD Pathway Bio-imager (BD Biosciences) as a percentage of Hoechst 33258-positive cells. Same procedure was performed with iNeurons treated with 10 μM glutamate (Sigma) for 8 hrs. LDH levels in the medium were measured as described in Material and Methods.

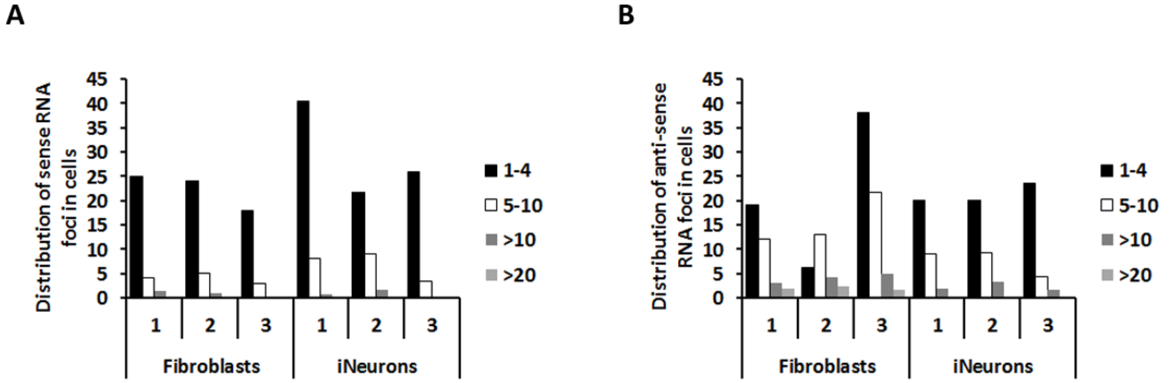

Supplementary Figure 1. Number of RNA foci per cell in fibroblasts and iNeurons. Distribution of (A) sense and (B) anti-sense RNA foci in the dermal fibroblasts and the corresponding iNeurons. Y-axes represent percentage of cells containing foci. Note the higher numbers of anti-sense foci per cell when compared to the sense RNA foci.

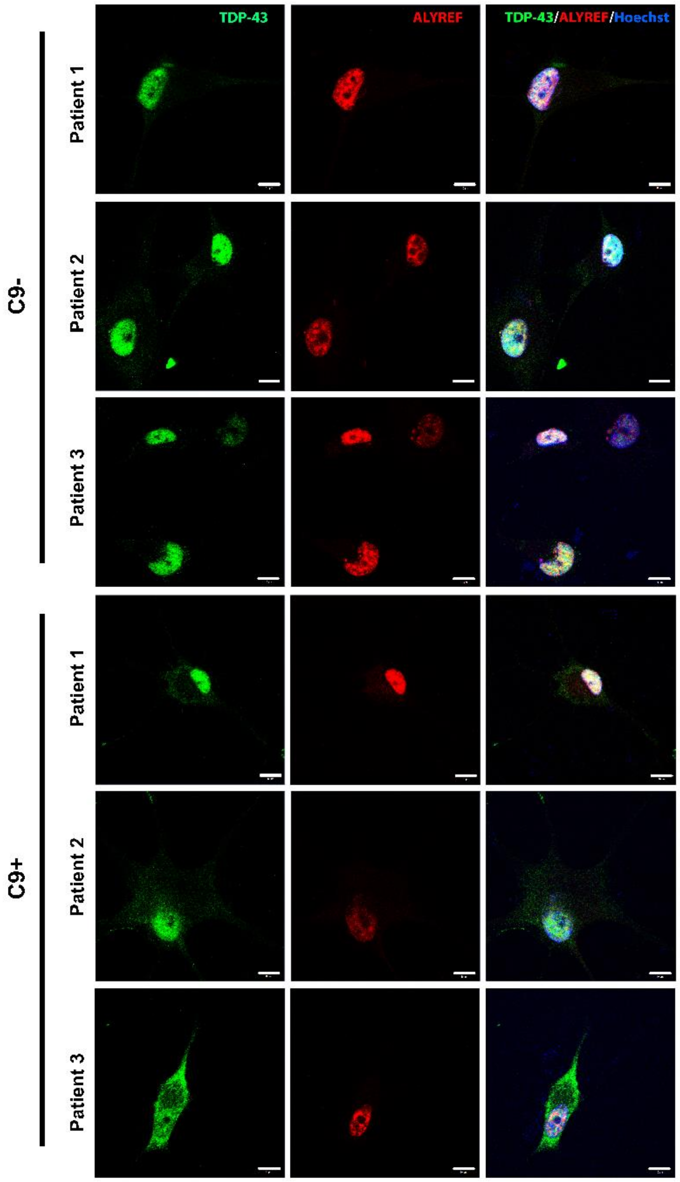

Supplementary Figure 2. TDP-43 localization in iNeurons. Cells were co-stained for ALYREF, a nuclear protein that has been shown to be sequestered by RNA foci in c9ALS/FTD neurons (Cooper-Knock et al., 2014). A partial translocation of TDP-43 to the cytoplasm was observed primarily in C9+ iNeurons. Blue staining: Hoechst 33258. Scale bars, 10 µm.

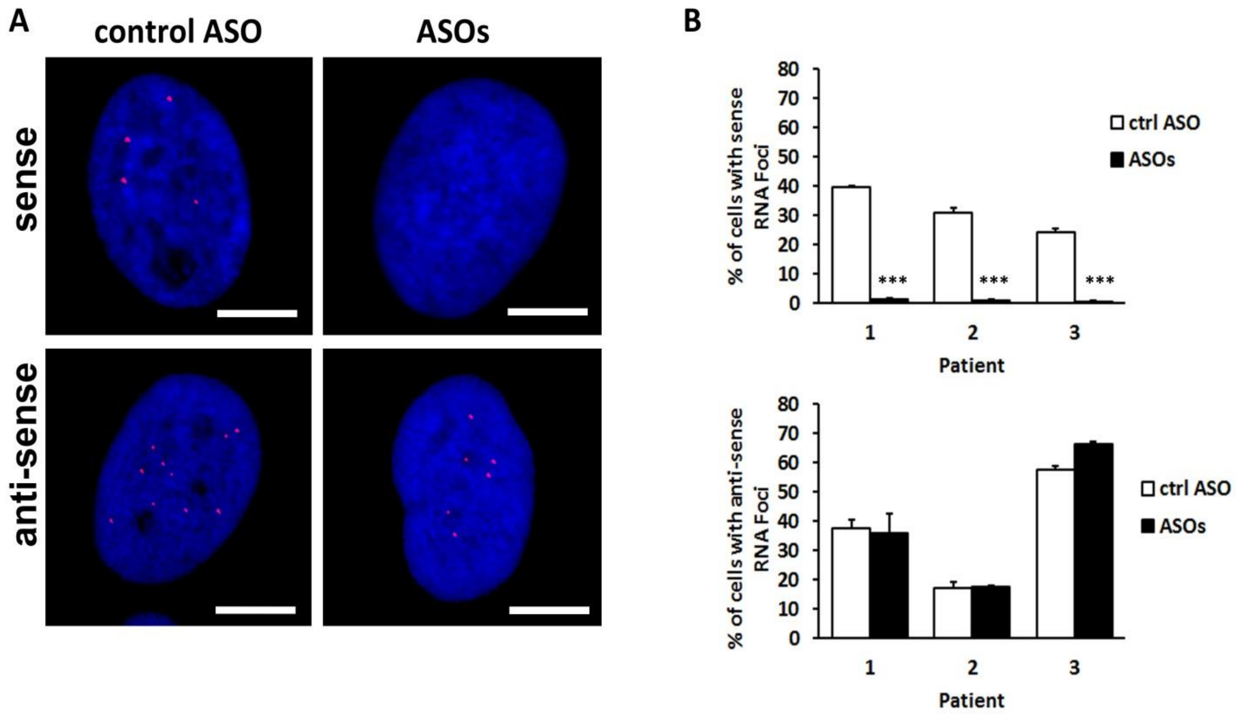

Supplementary Figure 3. Effect of ASOs on RNA foci in the fibroblasts. (A) ASOs reduce the number of C9+ fibroblasts with (S) but not (AS) RNA foci. Blue staining: Hoechst 33258. Scale bars, 20 µm. (B) Quantification of C9+ fibroblasts containing RNA foci. ****p*<0.001.

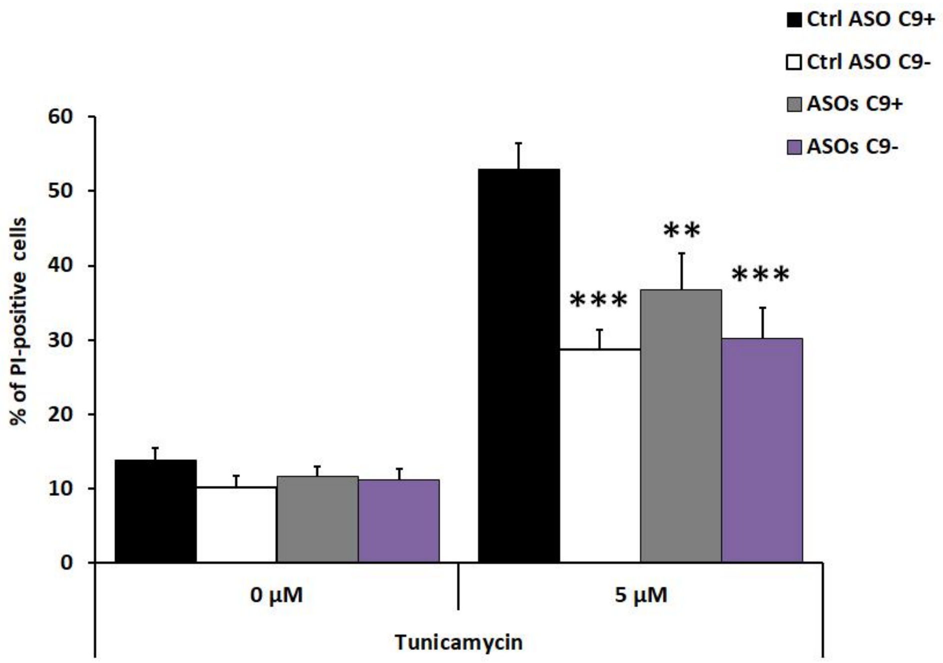

Supplementary Figure 4. Effect of anti-sense oligonucleotides targeting the sense strand of the *c9orf72* repeat, r(GGGGCC)exp, on cell viability (analyzed by PI staining) of iNeurons treated with 0 or 5 µM tunicamycin. ASOs reduce the tunicamycin toxicity in C9+ iNeurons. Data represents mean ± standard error of the mean (SEM; n=6, each with data from two wells, three C9+ and three C9- cell lines were used). (**$p<0.005$, ***$p<0.001$).

A

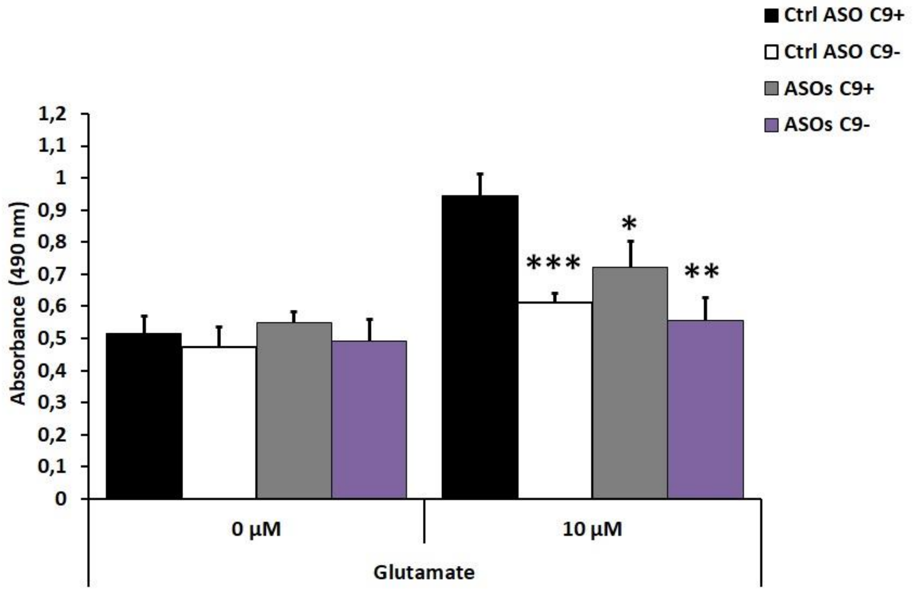

B

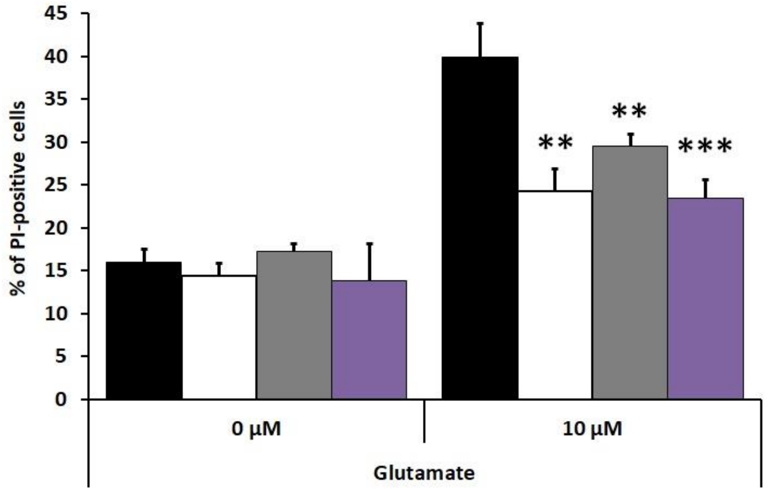

Supplementary Figure 5. Effect of anti-sense oligonucleotides targeting the sense strand of the *c9orf72* repeat, r(GGGGCC)exp, on cell viability of iNeurons treated with 0 or 10 µM glutamate. ASOs reduce the glutamate toxicity in C9+ iNeurons. (A) LDH assay. (B) PI staining. Data represents mean ± standard error of the mean (SEM; n=6, each with data from two wells, three C9+ and three C9- cell lines were used). (*$p<0.05$, **$p<0.005$, ***$p<0.001$).

Supplementary references

Cooper-Knock, J., Walsh, M. J., Higginbottom, A., Robin Highley, J., Dickman. M. J., Edbauer, D., et al. (2014). Sequestration of multiple RNA recognition motif-containing proteins by C9orf72 repeat expansions. *Brain* 137, 2040-2051. doi: 10.1093/brain/awu120
